# The Mycotox Charter: Increasing Awareness of, and Concerted Action for, Minimizing Mycotoxin Exposure Worldwide

**DOI:** 10.3390/toxins10040149

**Published:** 2018-04-04

**Authors:** Antonio F. Logrieco, J. David Miller, Mari Eskola, Rudolf Krska, Amare Ayalew, Ranajit Bandyopadhyay, Paola Battilani, Deepak Bhatnagar, Sofia Chulze, Sarah De Saeger, Peiwu Li, Giancarlo Perrone, Amnart Poapolathep, Endang S. Rahayu, Gordon S. Shephard, François Stepman, Hao Zhang, John F. Leslie

**Affiliations:** 1 National Research Council, Institute of Sciences of Food Production, (CNR-ISPA), via Amendola 122/O, 70126 Bari, Italy; giancarlo.perrone@ispa.cnr.it; 2 Department of Chemistry, Carleton University, Ottawa, ON KS5B6, Canada; david.miller@carleton.ca; 3 Department for Agrobiotechnology (IFA-Tulln), University of Natural Resources and Life Sciences, Vienna (BOKU), Konrad-Lorenz-Str. 20, 3430 Tulln, Austria; mari.eskola@boku.ac.at (M.E.); rudolf.krska@boku.ac.at (R.K.); 4 Institute for Global Food Security, School of Biological Sciences, Queen’s University Belfast, University Road, Belfast BT7 1NN, Northern Ireland, UK; 5 Partnership for Aflatoxin Control in Africa, Department of Rural Economy and Agriculture, African Union Commission, P.O. Box 3243, Roosevelt Street, Addis Ababa, Ethiopia; amarea@africa-union.org; 6 International Institute of Tropical Agriculture (IITA), PMB 5320, Oyo Road, Ibadan 200001, Oyo State, Nigeria; r.bandyopadhyay@cgiar.org; 7 Department of the Science of Sustainable Vegetable Production, Faculty of Agriculture, Food and Environmental Sciences, Universitá Cattolica del Sacro Cuore, via E. Parmense, 84-29122 Piacenza, Italy; paola.battilani@unicatt.it; 8 Food and Feed Safety Research, Southern Regional Research Center, USDA-ARS, 1100 Robert E. Lee Boulevard, New Orleans, LA 70124, USA; Deepak.bhatnagar@ars.usda.gov; 9 Departamento de Microbiología e Immunología, Facultad de Ciencias Exactas Físico-Químicas y Naturales, Universidad Nacional de Río Cuarto, Rutas 8 y 36, Km 601, Río Cuarto 5800, Córdoba, Argentina; schulze@exa.unrc.edu.ar; 10 Department of Bio-analysis, Faculty of Pharmaceutical Sciences, Ottergemsesteenweg 460, Ghent University, Gent 9000, Belgium; sarah.desaeger@ugent.be; 11 Oil Crops Research Institute, Chinese Academy of Agricultural Sciences, Xudong Second Road, Wuhan 430062, China; peiwuli@oilcrops.cn; 12 Department of Pharmacology, Faculty of Veterinary Medicine, Kasetsart University, Bangkok 10900, Thailand; fvetamp@ku.ac.th; 13 Department of Food Technology and Agricultural Products, Universiti Gadjah Mada, Yogyakarta 55281, Indonesia; endangsrahayu@ugm.ac.id; 14 Institute of Biomedical and Microbial Biotechnology, Cape Peninsula University of Technology, Symphony Way, P.O. Box 1906, Bellville 7535, South Africa; gshephard@mweb.co.za; 15 Platform for African-European Partnership in ARD, CTA Brussels Office, 39 rue Montoyer, 1000 Brussels, Belgium; fstepman@gmail.com; 16 State Key Laboratory for Biology of Plant Diseases and Insect Pests, Institute of Plant Protection, Chinese Academy of Agricultural Sciences, No. 2 West Yuanmingyuan Road, Beijing 100193, China; zhanghao@caas.cn; 17 Department of Plant Pathology, Throckmorton Plant Sciences Center, 1712 Claflin Avenue, Kansas State University, Manhattan, KS 66506, USA; jfl@ksu.edu

**Keywords:** consumers, education and outreach, food safety, food production, food security, health risks, trade, United Nations Sustainable Development Goals

## Abstract

Mycotoxins are major food contaminants affecting global food security, especially in low and middle-income countries. The European Union (EU) funded project, MycoKey, focuses on “Integrated and innovative key actions for mycotoxin management in the food and feed chains” and the right to safe food through mycotoxin management strategies and regulation, which are fundamental to minimizing the unequal access to safe and sufficient food worldwide. As part of the MycoKey project, a Mycotoxin Charter (charter.mycokey.eu) was launched to share the need for global harmonization of mycotoxin legislation and policies and to minimize human and animal exposure worldwide, with particular attention to less developed countries that lack effective legislation. This document is in response to a demand that has built through previous European Framework Projects—MycoGlobe and MycoRed—in the previous decade to control and reduce mycotoxin contamination worldwide. All suppliers, participants and beneficiaries of the food supply chain, for example, farmers, consumers, stakeholders, researchers, members of civil society and government and so forth, are invited to sign this charter and to support this initiative.

## 1. Introduction

Food safety is a pervasive concern of both the general public and of government authorities worldwide. Yet the insidious nature of mycotoxin contamination of key foods is frequently overlooked. Fungi are often found on crops growing in the field or in storage. Food-borne fungi are capable of producing hundreds of secondary metabolites but only a relative few are regulated, due to their adverse effects on human and animal health [1,2,3]. These metabolites include the widely regulated mycotoxins aflatoxins, fumonisins, tricothecenes (particularly deoxynivalenol), ochratoxins and zearalenone. Other mycotoxins that are less widely regulated include the ergot alkaloids, patulin and the T-2 and HT-2 toxins [4]. Mycotoxin exposures are not thought to directly affect human health in the developed countries of western Europe, Canada and the United States, although increased costs of food, especially for the most economically disadvantaged can alter purchasing decisions.

In developed countries, these problems generally are invisible but their management is an economic burden for producers, processors and consumers. These costs include the obvious ones of sampling and analysis to meet regulatory and contract requirements but also include larger, hidden costs of destroyed or returned shipments and the time and expense of sourcing and purchasing replacement items. Destroying non-compliant product, particularly when facing a recall is expensive in the short term because of the costs, frustration and time spent and often more expensive in the long term because of the loss of reputation of the company and country involved as a reliable supplier of high quality materials. Food importers often respond to these problems by creating new supply chains, often from different geographic regions [5].

The most information on economic impact is available for the United States but it is reasonable to anticipate that the information is broadly similar to agricultural economies in other developed countries. The cost of mycotoxin contamination to the U.S. economy was estimated to be between $2 and $3 billion per year depending on the year [6]. Testing is a considerable expense [7] with 10–20% of the projected loss spent to help ensure food safety. Millions of test kits are sold annually, official tests are required for only a fraction of the samples and costs of sampling and laboratory staffing often are not easily disaggregated from the costs of other operations. The majority of the costs of mycotoxin contamination are borne directly by farmers and are only indirectly passed along to consumers.

### 1.1. Codex Alimentarius, JECFA and EFSA

At the international level, comprising 187 member countries worldwide, the Codex Alimentarius Commission established by the Food and Agriculture Organization of the United Nations (FAO) and the World Health Organization (WHO) has adopted many food standards, guidelines and codes of practice applicable for mycotoxins in food and feed. These standards, guidelines and codes contribute to food safety and quality and to fairness in the global food trade. The Codex General Standard for Contaminants and Toxins in Food and Feed, adopted in 1995 and updated several times since then, lists maximum levels (MLs) for aflatoxins, deoxynivalenol, fumonisins, ochratoxin A and patulin in many different foods [8]. Also included in the Standard are protocols for sampling and performance criteria for the analytical methods used.

The Codex standards are based on the best available science and are regarded as neutral and generally accepted as an international standard. The Joint FAO/WHO Expert Committee on Food Additives (JECFA), as an independent scientific body, conducts risk assessments and provides scientific advice to the Codex. Several of JECFA’s human risk assessments of mycotoxins have informed the Codex texts. Codex standards are voluntarily applied by the members and form the basis for much but not all, national legislation. Codex codes of practice, such as the Code for the Prevention and Reduction of Mycotoxins in Cereal Grains and Grain-Derived Foods and Feeds, which was adopted in 2003, recommends practices that are based on Good Agricultural Practices (GAP) and Good Manufacturing Practices (GMP) and that are consistent with Hazard Analysis Critical Control Points (HACCP) principles [9].

As JECFA advises the Codex committee, so also does the independent agency European Food Safety Authority (EFSA) advise European risk managers, the European Commission (EC) and the European Union (EU) Member States. Similar entities, also advised by JECFA, advise other governments elsewhere in the world, for example, Health Canada and the US Food and Drug Administration. The numerous scientific mycotoxin risk assessments of EFSA form the foundation for EU mycotoxin legislation. EU maximum levels have been established not only for the same mycotoxin-food commodity combinations listed in the Codex but also for other mycotoxins and commodity combinations [10]. Regulations on mycotoxins in animal feed also are in force in the EU, as are the regulations for sampling protocols and performance criteria for analytical methods [11,12,13,14]. The EU legislation protects the public health and consumers’ interests and ensures the effective functioning of the EU market. Relevant portions of the EU legislation have been harmonized either completely or in large part with the Codex standards, guidelines and codes. The EU and all of its member states are members of Codex and work actively on mycotoxin issues to facilitate global trade of safe food and feed through the work of the Codex Committee on Contaminants in Foods.

### 1.2. Mycotoxins

#### 1.2.1. Aflatoxins

Aflatoxins, particularly their most abundant and toxic form, aflatoxin B_1_, are widely found in cereals (particularly maize) and groundnuts and are produced by several *Aspergillus* spp., the most prominent of which is *Aspergillus flavus*. Recently, the focus has been on aflatoxins produced in tropical and subtropical regions [15], although *A. flavus* also is commonly found in warm, temperate regions [16]. Global warming may expand the temperate regions in which aflatoxins are a persistent, rather than sporadic, problem. Aflatoxin B_1_ is a potent liver carcinogen in humans and is acutely toxic at high levels of exposure [1,17]. Aflatoxin exposure is associated with childhood stunting but the nature of the exposure required for stunting to occur is not well understood [18,19,20,21]. Mammals that consume dietary aflatoxin B_1_ convert it to aflatoxin M_1_, which is excreted in animal and human milk and is as cytotoxic as aflatoxin B_1_ [22] but has 10% or less of its carcinogenicity and mutagenicity [23]. In countries with chronic aflatoxin contamination of maize, animal production, most notably poultry production, is severely reduced, which leads to less protein in the diet and reduced milk quality [1,24].

Research on aflatoxin contamination in Africa now has a history of at least 50 years and there is a growing need to translate results obtained into applications to reduce the problem. More than 500 million Africans are still exposed at multiples of acceptable limits [17]. Poor awareness about aflatoxins, appropriate control measures to control contamination in the field and in storage and the negative health effects of aflatoxin consumption are reported in most African countries. Overdependence on grains in the diets of many Africans complicates the issue since these grains, usually maize, are both primary dietary components and common sources of mycotoxins. Successfully addressing mycotoxin exposure problems in these developing countries must go beyond mycotoxin detection and regulation to include more fundamental changes to and diversification of the diet consumed.

#### 1.2.2. Fumonisins

Fumonisins were first discovered in 1988 [25] and are a large family of compounds, of which fumonisin B_1_, B_2_ and B_3_ are of greatest concern, with fumonisin B_1_ being the most common in maize and maize products [26,27]. Fumonisins block ceramide synthase [28], an enzyme whose inactivation can result in many diverse metabolic changes [29]. Fumonisins are synthesized by a number of fungi, with *Fusarium verticillioides* and *Fusarium proliferatum* the most common problem in maize [30]. Fumonisins were first associated with equine leukoencephalomalacia, a brain disease in horses [31], but also are associated with pulmonary oedema in swine [32] and liver and kidney cancer in multiple rodent species and strains [1,17].

The role of fumonisins in human disease remains unresolved. The consumption of maize highly contaminated by fumonisin has been associated with high rates of oesophageal cancer in Italy and the former Transkei in South Africa [33]. Based on animal studies, fumonisin exposure may result in neural tube defects [1]. There also is good evidence that fumonisin exposure can result in childhood stunting [1,34,35,36].

#### 1.2.3. Trichothecenes

Trichothecenes [37] are a large group of mycotoxins, frequently found in maize and small grains such as wheat and barley in temperate regions worldwide. Deoxynivalenol, T-2 toxin and HT-2 toxin are the members of this class that have been the main concerns.

Deoxynivalenol, sometimes known as vomitoxin, is associated with contamination/infection by the fungus *Fusarium graminearum* and related fungi. This toxin was first described from infected wheat and barley in Japan in 1970, although disease epidemics in Japan back to at least the 1800s have been documented [38] and also are known to have occurred in China, Russia and India [24]. Deoxynivalenol is a mycotoxin that occurs widely, usually on small grains. High exposure levels lead to appetite suppression, feed refusal and emetic problems in animals and severe gastro-intestinal effects in humans [39,40,41], as the toxin affects intestinal, immune, endocrine and neurologic functions [40]. It is not considered to be a carcinogen [42]. Swine are the most affected domestic animal with losses in production resulting from refusal to consume grain contaminated at levels of ~1 mg/kg or higher [3]. Exposure to deoxynivalenol and its acetylated and modified forms is typically below the Provisional Maximum Tolerable Daily Intake (PMTDI) for adults in Europe, Canada, the United States, parts of South America and China, for example, [43,44,45,46] but may exceed these levels for children in some European countries [47,48,49,50]. At very high exposure levels deoxynivalenol has immunosuppressive properties but exposure to such high levels of deoxynivalenol usually are encountered only in laboratory experiments [51].

T-2 and HT-2 toxins also usually occur on small grains, but are less commonly detected than deoxynivalenol and related compounds. These toxins cause apoptosis by inhibiting protein synthesis, are immunosuppressive, and can cause blisters when they come in contact with skin [52]. Neither of these toxins is carcinogenic [53]. Both of these mycotoxins have been investigated as potential biological warfare agents [54] and are on the United States Select Agents and Toxins list [55]. T-2 is thought to be responsible for the large-scale deaths from alimentary toxic aleukia (ATA) in the first half of the twentieth century in the former USSR [55,56,57,58]. Regulation of subchronic exposure due to the immunotoxic and hematotoxic effects of T-2 and HT-2 is based on studies in mink, rats and swine [59].

#### 1.2.4. Zearalenone

Zearalenone was first recovered as a uterotrophic compound from fungal contaminated maize [60] and identified five years later as “F-2” [61]. Like trichothecenes, zearalenone is synthesized by *F. graminearum* and related fungal species. Zearalenone is not considered to be a carcinogen [42] and is of low overt toxicity [62]. It is, however, a potent oestrogen analogue and endocrine disruptor, with female pigs being the most sensitive domestic animal [63]. Zearalenone exposure at ~0.5 mg/kg can result in hyperoestrogenism and reduced reproductive capacity at lower exposures [3,62,64].

In humans, zearalenone has been associated with visible estrogenic effects in some case reports, although these reports are not always complete [65,66]. There is limited evidence supporting an association of zearalenone with idiopathic precocious puberty in populations in Italy, China and possibly the United States [67,68,69]. Although perhaps higher in the past [65], current levels of exposure for humans in developed countries generally are modest, see, for example, [62,64]. Zearalenone also occurs in some developing countries where environmental conditions favourable for the growth of *F. graminearum* can occur, for example, highland corn-producing areas in Africa [70,71]. The extent of human exposure to zearalenone in such areas warrants a more critical evaluation.

#### 1.2.5. Ochratoxin A

Ochratoxin A was first identified and characterized from fungal cultures in South Africa [72,73] and can be produced by several species of *Aspergillus* and *Penicillium*. It is a potent nephrotoxin in mammals [74] and a possible human carcinogen [75]. Ochratoxin A, is a common contaminant of cereal-based foods in temperate climates [76,77,78,79] and also can be found in diverse foods such as cacao [78,80], coffee [78,81], raisins [78,82], spices [78,83] and wine [78,84]. An outbreak of renal disease in swine in Scandinavia [85], first highlighted potential agricultural problems with this toxin. When first measured more broadly in the 1970s, ochratoxin A was very common in cereals sometimes at relatively high levels [24]. Based on the well-understood renal toxicity, regulation of ochratoxin in Europe in 2006 has been very successful at lowering overall exposure [77].

#### 1.2.6. Patulin

Patulin [86] is a mycotoxin produced by several species of *Aspergillus*, *Byssochlamys* and *Penicillium*. It is most commonly recovered as a contaminant of fruit juices, particularly apple juice and its presence may be used to estimate the quality of apples or juice. Patulin does not survive the fermentation of apple juice to cider [87]. This toxin has been described under a number of different names, including clavacin, claviformin, expansin, mycoin c and penicidin [88], since the 1940s [89,90]. Although initially of interest for its antibiotic properties [91], testing was halted due to undesirable side effects. The most recent JECFA evaluation [92] confirmed patulin’s association with gastro-intestinal problems. These problems are likely attributable to the toxin’s ability to bind sulfhydryl groups, thereby inhibiting the activity of many enzymes. Patulin is not considered to be a carcinogen [93].

#### 1.2.7. Ergot Alkaloids

Compounds in the ergot alkaloid class [94], commonly found in cereals such as wheat, rye, oats and barley, are synthesized primarily by fungi in the genus *Claviceps*, although the link between ergot and a fungal source was not well understood until the mid-1700s. Consumption of these toxins can result in vasoconstriction at the extremities and loss of a limb, for example, hand, foot, finger or toe, accompanied by a burning pain in the affected area often termed “holy fire” or “St. Anthony’s fire.” Alternatively, ergot alkaloid exposure may result in delirium, hallucinations, muscle spasms, diarrhoea and convulsions. These symptoms of mycotoxicoses have been well documented in humans since 944 A.D. in Europe and probable cases have been identified in China, Assyria, Sumeria and Mesopotamia between 1000 and 2000 B.C. [95].

A number of other fungal secondary metabolites are recognized as potentially problematic [96] and some are likely to be regulated in the near future.

#### 1.2.8. Co-Exposure

Laboratory studies usually focus on the effects of a single toxin, or group of related toxins, on a single target species. Outside the lab, exposure to multiple toxins in the same food is possible and in some locations common. The co-exposure usually envisioned is to aflatoxin B_1_ and fumonisins, although aflatoxin M_1_ cytotoxicity also is known to increase in the presence of ochratoxin A and zearalenone [97]. In much of Africa as well as parts of Central America, co-exposure to both aflatoxin and fumonisin is common [1,17,98]. In laboratory animals, co-exposure suggests an additive or synergistic effect of fumonisin and aflatoxin in the development of hepatocellular carcinoma [1,99] and possibly increased immunotoxicity and reduced growth of some cell lines [98]. There are no data demonstrating an interaction between aflatoxin and fumonisin in humans, although survey reports suggest both additive and synergistic interactions may occur [100]. As noted by the 83rd JECFA [1], “The interaction between aflatoxin B_1_, a compound with known genotoxic properties and fumonisins, which have the potential to induce regenerative cell proliferation (particularly at exposures above the PMTDI), remains a concern.” Definitively determining interactive effects in humans will be difficult given the complexity of most diets and the inconsistent and confounding toxin contamination in many naturally occurring foodstuffs, especially in developing countries.

### 1.3. Development of the Charter

The United Nations Environment Programme (UNEP) and the WHO International Programme on Chemical Safety (IPCS) have both declared that humans have a right to food free from mycotoxins that could cause significant health risk. Nonetheless, mycotoxins in staple crops remain a significant foodborne risk to human health, animal health and market access [3]. Analyses by the United Nations Education Program and others note that climate variability is increasing the risk for mycotoxin contamination in some areas [101,102,103]. Much attention has been paid by the European Commission and the Chinese government to mycotoxin issues due to mycotoxin contamination in agricultural products being sold by China to the EU. Horizon 2020 programs on mycotoxin issues mandated interactions between European and Chinese partners. Both MycoKey [104] and MyToolBox [105], the two Horizon 2020 programs that focus on mycotoxins, are expected to provide innovative integrated solutions for sustainable mycotoxin management along numerous food and feed chains in both developed and developing countries. International interdisciplinary research in mycotoxins remains a dynamic area, with general directions from a series of round table discussions now available [106]. During the first 18 months of the MycoKey project, the idea of a Mycotox Charter was discussed and a draft document developed. The MycoTox Charter is also a response to the demand for further action in this area engendered by previous European Framework Projects, that is, MycoGlobe and MycoRed. More recently, a web site [107] was established to enable people and institutions worldwide to contribute to the discussion about increased equity in the availability of safe food.

## 2. Mycotox Charter Structure

The Charter is structured in sections that delineate the rights, awareness and commitments of its supporters. The major sections are:

### 2.1. Preamble

The primary purpose of the Charter is to provide a globally applicable statement for food and feed suppliers and consumers on mycotoxin control and reduction that enhances food and feed safety along the supply and consumption chains. Sharing and spreading common intentions and behaviours can sustain and boost collective action towards this goal.

### 2.2. Rights

All people have the right to access a sufficient quantity of safe, healthy, nutritious food, that is, with mycotoxin content below regulatory limits, at an affordable price.

### 2.3. Awareness

Mycotoxin contamination is a longstanding problem in public health and represents a great challenge for at least the next decade. Mycotox Charter supporters are aware of the economic and health risks arising from mycotoxin contamination in agricultural crops and other food products. This awareness needs to be increased and broadened. Consumers need to become more aware of the health risks posed by exposure to mycotoxin-contaminated food and empowered to demand safe, quality food on a routine basis.

### 2.4. Commitments

As signatories to the Mycotox Charter, supporters accept responsibility for leaving a healthier, fairer, more sustainable world to future generations and commit to increasing food safety, fostering responsible and sustainable consumer behaviour and providing recommendations for mycotoxin control and regulation. They embrace the principles and practices outlined below in the Mycotox Charter, which will reduce mycotoxin contamination in food and associated health hazards and contribute to the achievement of United Nations Sustainable Development Goal Nos. 2 and 10 [108].

## 3. Mycotox Charter Declaration

We, the undersigned endorse this Mycotox Charter and make a clear commitment to everyone’s right to sufficient safe food, to minimization of mycotoxin contamination and to appropriately established food laws that protect public health and that safeguard trade of food and feed. This commitment is fundamental to minimizing the differences in access to sufficient safe food for all humanity.

We believe that only our collective action as citizens, together with businesses and local, national and international institutions, will enable us to overcome the major challenges posed by mycotoxin contamination and to improve food safety at a global level. These challenges include:

- Insufficient awareness of mycotoxin occurrence, accumulation and metabolization;
- Slow and often expensive analytical methods for detecting mycotoxins at regulatory levels;
- Minimizing the negative impact of mycotoxin exposure, including undernutrition and malnutrition, on human and animal health;
- Reducing waste of mycotoxin-contaminated food and feed;
- Ensuring sustainable management of mycotoxins in food and feed production processes;
- Developing effective food safety policies, including mycotoxin regulations, in countries that lack such policies and, often, the functional institutions that can effectively disseminate and enforce them.

### 3.1. In Signing the Mycotox Charter:

- We affirm our collective responsibility to implement practices and choices that lead to the reduction of mycotoxins in food and feed and to increased food safety for future generations;
- We commit to advocate for decisions that realize the fundamental goal of harmonizing regulations that impact mycotoxin exposure at the global level, with particular attention given to countries where regulations are poorly developed or non-existent and thereby enable more equitable access to safe food and feed worldwide.

### 3.2. We Recognize That:

- Mycotoxins are major food contaminants that reduce global food safety and security and may be especially important in developing and middle-income countries;
- Everyone has the right to access, at an affordable price, a sufficient quantity of safe, healthy and nutritious food, with mycotoxin content as low as reasonably achievable;
- Mycotoxins remain a “largely ignored global health issue” [109];
- “There is a lack of action to address the problem of mycotoxins mostly in low-income countries and the reasons for it are undoubtedly complex and incompletely researched” [109] and that this problem has persisted for decades [110];
- Food spoilage caused by mycotoxins affects a staggering amount of the world’s food supply and increases food insecurity;
- Mycotoxin contamination is a major non-tariff trade barrier for agricultural products, which negatively impacts the health and income of small-holder farmers, regional and international trade and the world economy;
- More than one mycotoxin may co-occur in feeds and food products, leading to joint exposure with health impacts that are not clear and may be additive or synergistic;
- New and emerging/re-emerging mycotoxins [96] have been reported in some food crops and may create additional problems;
- Greater dietary diversification and less dependency on specific crops is crucial to reduce the level of mycotoxin exposure;
- GAP and GMP, including HACCP principles, are important strategic measures to address mycotoxin contamination;
- Improved storage facilities, especially at the farmer and small trader level, will reduce fungal growth and mycotoxin contamination in stockpiled commodities;
- Early warning of mycotoxin contamination can limit the extent of damage and more readily enable remediation;
- All of these challenges are greater in countries that are food insecure [17].

### 3.3. We Consider It Unacceptable That:

- Practices to reduce mycotoxin contamination exist, yet the resources and efforts available to implement these practices can be insufficient at multiple points along the food chain [111];
- Inspection, regulatory and enforcement capabilities often are inadequate, especially in developing countries and when applied are not applied uniformly;
- Informal and dispersed markets often lack controls, which can increase mycotoxin exposure risks;
- High quality food may be exported leaving poorer quality food to be consumed domestically with the poorest members of society often exposed to the highest levels of mycotoxins;
- There are unjustifiable inequalities in the opportunities for people to access food that contain levels of mycotoxins below that considered safe for human consumption;
- People suffering from chronic hunger, who are malnourished, or have stunted growth also are exposed to higher levels of mycotoxins;
- Each year, tens of thousands of tonnes of food produced for human consumption are wasted due to high levels of mycotoxin contamination.

### 3.4. We Are Aware That:

- The economic losses and health hazards posed by mycotoxin contamination of food and feed are a huge challenge;
- Climate change could have an important impact on mycotoxin contamination of food and feed and these changes could disproportionally affect economically disadvantaged people;
- Mycotoxin regulations and technological advances can help manage risks posed by mycotoxin contamination;
- Monitoring well-known mycotoxins in regularly consumed foods and feeds may become easier as technology advances but the identification of new and emerging mycotoxins may complicate monitoring, food and feed safety evaluations and risk assessment;
- Mycotoxin contamination is a longstanding public health issue and exposure to mycotoxins in daily diets can pose a health risk for humans and domesticated animals;
- Infants and children are particularly susceptible to the adverse health effects that follow mycotoxin exposure;
- Animal welfare and production are affected significantly by exposure to subclinical levels of mycotoxins;
- The lack of biomarkers for assessing exposure of humans and animals to many mycotoxins limits our understanding of the health effects resulting from mycotoxin exposure.
- Risk is multi-source, with different foods posing different risks, with food safety most effectively addressed from a dietary rather than a commodity perspective;
- In Africa, grains, commonly maize, provide nearly half the daily energy intake, perhaps more in rural areas and this over dependence on grains for nutrition increases mycotoxin exposure significantly beyond what is observed in most developed countries;
- Improved consumer education on healthy diets and safe food storage and preparation methods can reduce mycotoxin exposure;
- Improvements in knowledge of and practical experience with mycotoxin control and reduction in both traditional and advanced food and feed production systems are critical in both developed and developing countries;
- Any intervention(s) to reduce mycotoxin contamination and the consumption of contaminated food and feed require a sound and thorough understanding of the markets in which the contaminated food and feed are traded;
- Cultural changes can be difficult to implement and the research and development communities must understand potential non-monetary incentives and impediments to needed changes for improvements to be adopted and implemented.

## 4. Mycotox Charter Commitment

### 4.1. Since We Are Responsible for Leaving a Healthier, Fairer, More Sustainable World to Future Generations, as Citizens, We Commit to:

- Improve the safety of available food and feed, particularly by reducing its mycotoxin content;
- Provide advice on mycotoxin regulations developed by academic and governmental experts and their practical implementation;
- Promote education in the family context of dietary and environmental factors and choices that affect mycotoxin exposure;
- Provide farmers with information that enables rational decisions/solutions for addressing potential mycotoxin problems;
- Respect and protect the environment through responsible behaviour and sound practices, such as recycling or remediation, to reduce mycotoxins in food and feed;
- Play an active role in building a sustainably safe food supply, with lower mycotoxin content, including innovative solutions, that stem from our collective work, creativity and skills.

### 4.2. As Members of Civil Society, We Commit to:

- Make our voices heard at all decision-making levels—local, national and international—relevant to mycotoxin issues;
- Represent civil society bodies in debates and processes that formulate public policy and shape public opinion on mycotoxin exposure and risk management;
- Increase the interest of the scientific community in developing countries and the research conducted there on mycotoxins;
- Increasing human resource capacity devoted to mycotoxicology;
- Strengthen and supplement an international network of projects, actions and initiatives on mycotoxins as related to food safety and security;
- Identify and report critical issues in international regulation of and standards for mycotoxins;
- Share new knowledge and technologies with all countries to enable them to meet and adopt the most current international mycotoxin regulations and standards.

### 4.3. As Businesses, We Commit to:

- Increase access to quality food for all by considering the limitations of producers and designing cost-effective strategies to minimize mycotoxin exposure;
- Improve product safety and enhance profitability for all participants in the food chain, while minimizing price increases for consumers;
- Develop new strategies to monitor and predict mycotoxin contamination in specific foods/feeds and in particular geographic regions;
- Sustain the systematic development of research centres that work on mycotoxins;
- Promote the establishment of national and regional hubs of excellence that focus on mycotoxins of local interest and that can provide locally relevant information in response to mycotoxin contamination outbreaks;
- Invest in research, promoting a wider sharing of results and their implications for the collective good, without distinction between the public and the private sector;
- Build a database of information on health-related risks associated with mycotoxins;
- Translate existing monitoring technologies into on-site/storage-specific application tools for growers to record data on mycotoxin contamination and to communicate these data through modern information and communication technology (ICT) systems;
- Improve production, storage, processing and logistics, to reduce (or eliminate) mycotoxin contamination;
- Produce and market healthy, safe food, informing consumers about the mycotoxin risk;
- Contribute to achieving the United Nations Sustainable Development Goals [108], by using innovative processes, products and services and by adopting and practicing codes of social responsibility [112].

### 4.4. Through our Signing of the Mycotox Charter, We Strongly Urge Governments, Institutions and International Organizations to Commit to:

- Include mycotoxin control in national strategies and plans to protect public health and in relevant long-term development projects, to establish priorities and to implement sustainable actions;
- Harmonize risk-based regulations and standards governing mycotoxin exposure to increase food safety and food security and to make these standards and regulations more effective;
- Invest in infrastructure and training to develop local and national risk management capacity and thereby increase/enable effective, equitable implementation of mycotoxin regulations and standards;
- Highlight in international forums the role of mycotoxin contaminated food in nutrition/ malnutrition to ensure that the problem receives due attention at the national level and that responses to the problem are coordinated amongst specialized international organizations;
- Formulate and implement rules, regulations and standards regarding mycotoxins in food, feed and environmental safety that are easy to understand and effective when applied;
- Promote the culture of a safe, diverse, healthy diet with low mycotoxin exposure as a global health tool;
- Develop national health service measures and policies that promote a healthy, sustainable diet that minimizes mycotoxin exposure for people with special nutritional requirements;
- Increase resources for research on mycotoxins and the dissemination of the results, especially in geographic regions where consumer risk of significant mycotoxin exposure is high;
- Increase education on and facilities for GAP and GMP for farmers and small-scale industries and integrate these practices into mycotoxin management systems that can be readily adopted at critical points along the entire food chain from farmer to consumer.

We believe in the possibility of a world with reduced risk of mycotoxin contamination and without chronic suffering from the adverse health consequences resulting from exposure to mycotoxins. We commit to adopting the principles and practices outlined in the Mycotox Charter as a strategy that all countries should follow to reduce the problem of mycotoxins in food and related health hazards. The importance of the reduction of dietary exposure to mycotoxins is evident from the number of interdisciplinary EU and international projects funded on mycotoxin research.

By signing the Mycotox Charter, we declare our concrete and active support for the Sustainable Development Goals promoted by the United Nations [108]. We encourage readers to go to the web site for the Charter [108] and to add their support to ours.

## 5. Conclusions

Considering the high risk of mycotoxin exposure associated with the consumption of some foods, it is essential to strengthen food safety measures. We think this strengthening can best be done by increasing awareness of consumers, policymakers and businesses of potential mycotoxin dangers and by global adoption of GAP, GMP and HACCP principles and practices designed to reduce mycotoxin exposure. Strengthening an international network of projects, actions and initiatives on mycotoxins and food safety, harmonizing worldwide regulations and standards based on mycotoxin exposure, enhancing education and outreach efforts and supporting more equitable access to a safe, nutritious, diversified diet, are fundamental goals to be pursued by producers, processors, researchers, consumers, politicians and governments and are all captured by the spirit of the Mycotox Charter.
